# Essay on Solid Intra-Thoracic Tumours

**Published:** 1846-07

**Authors:** 


### Art. XXI.

*Essai sur les Tumeurs Solides Intra-Thoraciques.* Par J. M. H. Gintrac. Thèses de Paris, Janv. 1845.

*Essay on Solid Intra-Thoracic Tumours.* By J. M. H. Gintrac, &c. &c. 4to, pp. 67.

In this Essay, such tumours as are formed within the walls of the chest, and yet without the proper structure of the organs that cavity contains, are treated of. The author has succeeded in collecting thirty-two cases of the kind. The nature of the material composing the new growth in these cases varied, being encephaloid, scirrhous, tuberculous, steatomatous, &c. This variety of component material he does not regard as a fair ground for separating the cases, because various kinds of structure sometimes appear in one and the same tumour, because the distinctions between these various kinds of structure are not always clearly defined, and because their local effects and ultimate influence are frequently similar. Hence the only division adopted by the author is one of situation as follows: 1, tumours developed in the pleuræ; 2, outside the pleural sacs, either between the lung and pleura, the ribs and pleura, or in the mediastina; 3, tumours connected with the bones of the thorax.

Passing over the narratives of the cases, transcribed (with one exception) from various published works, we reach the writer's general considerations on these growths.

In respect of causes, we learn that these productions are rare in infancy, youth, and old age; most common in adult age. There were nineteen men for nine women. The disease occurred in persons of all varieties of constitution, profession, and previous health; local injury appeared in a few instances, as did also some general causes, to have determined or accelerated the outbreak of the disease.

The analysis of the symptoms includes dyspnœa (the most constant of all); cough, which was common; expectoration, variable in nature or amount; and pain of the thorax, varying in character and in exact site. Inspection of the thorax sometimes discovered tumour protruding externally. "Percussion was in general dull in the regions where the tumours were seated. Auscultation disclosed absence or diminution of the respiratory murmur on the affected side. Mucous or crepitant râles have been observed, and in a case of sub-sternal tumour, the 'souffle cataire' was heard." This is absolutely the total amount of what is said on the subject of physical signs! We cannot avoid contrasting this ludicrously meagre notice of this most important branch of the author's subject with the minute and elaborate investigations and descriptions of these same physical signs by Dr. Walshe, in the sections on Cancer of the Lungs and Mediastina, in his recent work. While those sections furnish manifest proofs that the number and variety of physical signs is greater in cases of intra-thoracic tumour than of almost any other affection of the respiratory organs, they supply pleasing evidence of the superior advancement of our countrymen in sound acquaintance with this class of diseases.

Palpitation of the heart existed in some cases, attended in one instance with blowing murmur; the heart was pushed to the right or left, according to the seat of the tumour. The pulse varied in character. The face was sometimes flushed or livid; in other cases pale or infiltrated. The lower, and sometimes the upper, limbs were œdematous; the œsophagus, diaphragm, stomach, and liver, underwent pressure in various cases;—hence dysphagia, hiccup, vomiting, swelling of the hypochondria, and jaundice. The appetite held good till an advanced period; the state of the bowels varied.

The course of the disease, though generally equable, was sometimes intermittent. Its duration was scarcely capable of being calculated; death sometimes occurred suddenly.

There is nothing worthy of particular notice in the writer's observations on the diagnosis.

In the hope of effecting the resolution of intra-thoracic tumours, the author recommends a local discharge to be kept up by caustic issues. He would wish an abundant suppuration produced by the successive production of eschars in various parts of the chest. We doubt exceedingly the value of such applications as curative agents: it is probable they might relieve the dyspnœa and pain, if made to act very energetically; but it would then become a question (and in our minds the question should at once be answered in the affirmative) whether the remedy were not worse than the disease. The iodide of potassium is recommended, *more solito* —still we believe that something in the way of suspended progress may fairly be hoped for from the action of iodine well managed, and given internally and applied externally. The extracts of conium, hyoscyamus, belladonna, and aconite are thought worthy of trial by the writer. Blood-letting may be required for the relief of urgent dyspnœa and symptoms of congestion of various parts and organs; but the writer correctly insists on the importance of being cautious with the lancet: a symptom may be relieved by its use, but the activity of the main disease certainly does not seem to undergo (and why should it?) the very slightest favorable modification by abstraction of blood, local or general. The author seems an advocate of the *cura famis,* but he is not one of the ultra starvationists. It must be a strong faith indeed in the powers of starvation as a remedial agent, which would induce the physician to deprive of food the wretched sufferer, whose appetite is unimpaired, and who, having little but his own woes and apprehended death to dwell upon, turns to the comfort of satisfying hunger as his sole earthly indulgence,—as to the "*dernier fil auquel tient le bonheur d'exister.*" Probably M. Gintrac has not yet treated many such cases as these; when the sufferers become his patients, he will, we predict, find some difficulty in enforcing the *cura famis.*

---

